# The Library of the Physician

**Published:** 1894-05-19

**Authors:** 


### The First Century of English Medical Literature.

III.—THE LIBRARY OF THE PHYSICIAN.

It would be interesting to know the exact course of study considered necessary for a physician in the days of Henry VIII. No course of clinical instruction was open to him, and his opportunities for gaining knowledge of the symptoms of diseases for which he was to prescribe were no doubt very limited. Nor was there much encouragement probably given in the faculty to the youth who formed his judgments on observation. Authority was all in all, and so fixed had become this principle in medicine that even the new school which towards the end of the sixteenth century had increased in importance, relied no less blindly on the teaching of Bombast and his immediate followers than the more conservative professors did on the dictates of Galen and Avicenna.

Sir Thomas Elyot, Knyght, who in the year 1541 brought out his "Castle of Health" in English, gives an instructive account of his own studies in medicine: "Before that I was twenty years old, a worshipful physician and one of the most renowned at that time in England, perceiving me by nature inclined to knowledge, read unto me the works of Galen, of temperaments and natural faculties, the Introduction of Johannicius, with some of the Aphorisms of Hippocrates. And after of my owne study I read over in order the works of Hippocrates, Galen, Oribasius, Paulus Celius, Alexander Trallianus, Celsus, Plinnis, the one and the other, with Dioscorides, nor did I omit to read the long canons of Avicen, the commentaries of Averrois, the practice of Isake, Halyabbas, Rasys, Mesue, and also of the more part of them which were their aggregatours and followers. And although I have never been at Montpellier, Padua, nor Salern, yet have I found something in physic whereby I have taken no little profit concerning mine owne health." This list of authorities gives a fair idea of the extent of the studies of the average physician of that time. A tolerable working knowledge of Latin is pre-supposed, since most of these works were then only accessible in that language. It will be seen that English authorities there were none, and his most modern author is the Dutch Isaac, who flourished in the thirteenth century.

But a new spirit was even then abroad. The cloak of mystery which had been studiously folded round matters medical by its professors was, in the course of the next fifty years, to be cautiously and apologetically stripped off by numerous busy translators, and the result was to give an enormous impulse to the science, even if it fed the indiscretion and curiosity of the layman.

The most important contributions secured to medicine by the translators were undoubtedly the works of the modern Continental physicians. The ancients are represented in these early Black Letter volumes only in fragments in strangely distorted guise, after passing through more or less erroneous French and Latin versions. The earliest translation of Galen, for instance, is that of Robert Copland, known in his day as the oldest printer in London. He calls it "The fourth Boke of the Terapeutyke or Method curatyfe of Claude Galyen, prynce of Physiciens," and it was "newly translated out of the frenshe at the instigacion and costes of the ryght honest parson, Henry Dabbe, stacyoner," November 7th, 1541. Later on certain works of Galen were translated by J. Jones (De elementis, 1574); by George Baker (De compositione medicamentorum, 1574); by Thomas Gale (Methodus Medendi, 1586); and by John Read (Dia Chalciteos, 1588). Quaint and almost indecipherable versions of the Aphorisms and Prognostications of Hippocrates and of the Sayings of Celsus were also issued. They can hardly have been intended for the physician's library.

It was a strange thing, abhorent to many, to see any work on medicine in English. George Baker, surgeon to Queen Elizabeth, and master of the Barber Surgeons' Company, expresses the feeling of many in his preface to the "Newe Jewell of Health," which he translated from Conrad Gesner. He is deeply conscious of the adverse criticism he will invite by issuing a work intended for the profession in his native tongue. At least he has left the names of the simples in the Latin as they were, for he pleads, "I would not have every ignorant asse to be made a chirurgian by my book, for they would do more harm with it than good." The book in question represents perhaps the highest point to which the study of medicine had then been carried, and must have been indispensable on the shelves of the Elizabethan physician, who had already shot far ahead of Sir George Elyot's somewhat musty collection. Gesner, who died in 1565, was a man of vast erudition, an excellent botanist and naturalist, and as a physician acquainted with the use of many remedies to which a far later origin has been attributed. He was specially successful in his use of hellebore, of opium, of linseed oil in pleurisies, and of cold water in acute diseases. The book which Baker translated treated of the manner of making distillations, extracting salts and preparing antimony and potable gold. It also gave directions for the making of the vessels, furnaces, and instruments necessary in these operations for which the doctor himself was in early days responsible, since the apothecaries rarely undertook more than the sale of the simple ingredients. But the delicacy of many of these processes rendered them quite impracticable to any except an expert, and gave rise before long to a new order of distillers, or as they preferred to call themselves, "spagericks," who approximated to the modern dispensing chemist.

George Baker mentions several of these distillers with approbation. By far the most famous was John Hester, of Paul's Wharf, who was himself a busy translator as well as preparer of drugs, and whose works were chiefly acceptable in all probability to the doctor who valued the "newest lights." He was an enthusiastic admirer of Paracelsus, whom he introduced to the English reader in a translation through the German of "A Hundred and Fourteene Experiments and Cures," 1596, dedicated to Walter Raleigh, Esq. He combined with this tractate translations from Barnard Londrada a Portu Aquitanus, from Isaac Hollandus, and from Quercetanus, the result being a very heterogeneous mixture of the new and the old. But he confesses in his dedication that he has translated them "not for their method, which I meddle not with, but for their medicines, which I usually make." This seems to have been the temptation for his translating the "Rational Secrets" of Fioravanti, a contemporary practitioner of Bologna, who, however successful he may have been as a surgeon, was frankly empirical in his medical theories. John Hester's original work, "The Pearle of Practise," published by his friend Fourestier in 1394, after his death, is, as might be expected, a mere collection of miscellaneous remedies and reputed cures. He marks an important stage in English medicine, however, when the value of certain foreign discoveries was beginning to be felt, and men were eager to study and imitate the most fantastic experiments.

